# Retrospective analysis of the frequency of centrofacial telangiectasia in systemic sclerosis patients treated with bosentan or ilomedin

**DOI:** 10.1186/2047-783X-19-2

**Published:** 2014-01-10

**Authors:** Sonja Hetzer, Bettina Alexandra Buhren, Holger Schrumpf, Edwin Bölke, Stephan Meller, Kai Kammers, Peter Arne Gerber, Bernhard Homey

**Affiliations:** 1 Department of Dermatology, Medical Faculty, University of Duesseldorf, Moorenstrasse 5, D-40225 Duesseldorf, Germany; 2 Department of Biostatistics, Johns Hopkins Bloomberg School of Public Health, Baltimore, MD 21205, USA

**Keywords:** Endothelin, Bosentan, Iloprost, Telangiectasia, Systemic sclerosis

## Abstract

**Background:**

Bosentan is a dual endothelin receptor antagonist initially introduced for the treatment of pulmonary arterial hypertension and recently approved for the treatment of digital ulcers in patients with systemic sclerosis (SSc). Our clinical observations indicate that bosentan therapy may be associated with an increased frequency of centrofacial telangiectasia (TAE). Here, we sought to analyze the frequency of TAE in patients with SSc who were treated with either bosentan or the prostacyclin analog iloprost.

**Methods:**

We conducted a retrospective analysis in 27 patients with SSc undergoing therapy with either bosentan (n = 11) or iloprost (n = 16). Standardized photodocumentations of all patients (n = 27) were obtained at a time point ten months after therapy initiation and analyzed. A subgroup of patients (bosentan: n = 6; iloprost: n = 6) was additionally photodocumented prior to therapy initiation, enabling an intraindividual analysis over the course of therapy.

**Results:**

After ten months of therapy patients with SSc receiving bosentan showed a significantly (*P* = 0.0028) higher frequency of centrofacial TAE (41.6 ± 27.8) as compared to patients with SSc receiving iloprost (14.3 ± 13.1). Detailed subgroup analysis revealed that the frequency of TAE in the bosentan group (n = 6 patients) increased markedly and significantly (*P* = 0.027) by 44.4 after ten months of therapy (TAE at therapy initiation: 10.8 ± 5.1; TAE after ten months of therapy: 55.2 ± 29.8), whereas an only minor increase of 1.9 was observed in the iloprost group (n = 6 patients; TAE at therapy initiation: 18.3 ± 14.5; TAE after ten months of therapy: 20.2 ± 15.5), yet without reaching statistical significance (*P* = 0.420).

**Conclusions:**

The use of bosentan may be associated with an increased frequency of TAE in patients with SSc. Patients should be informed about this potential adverse effect prior to therapy. Treatment options may include camouflage or laser therapy.

## Background

Scleroderma (systemic sclerosis, SSc) is a rare autoimmune disease characterized by excessive extracellular matrix deposition, fibrosis and vascular alterations [1,2]. The disorder can affect almost any organ, including the kidneys, the gastrointestinal tract, lungs or heart, and most notably the skin, and may lead to severe dysfunction up to complete organ failure [3]. The two major forms of SSc are localized scleroderma and systemic scleroderma. Localized scleroderma is the more common form of the disease and only affects the skin without any internal organ involvement. By contrast, systemic scleroderma or systemic sclerosis is characterized by cutaneous and non-cutaneous involvement and can be further subdivided into limited cutaneous scleroderma (lcSSc) and diffuse cutaneous scleroderma (dcSSc). The latter types are defined with regard to the extent of skin tightening, the number of affected inner organs as well as their typical autoantibody profile. Any combination of SSc and a rheumatologic disease such as lupus erythematosus, polymyositis, rheumatoid arthritis or Sjögren’s syndrome, is referred to as overlap syndrome [2-4].

Clinically, digital ulcers and gangrene are a frequent and chronically recurrent complication of SSc and may result in considerable disability [5-7]. Of note, the incidence for finger amputation was reported to be as high as 1.2% per patient-year in patients with SSc affected by digital ulcers [5]. The main causes of digital ulcers are SSc-associated vascular alterations [8,9]. Vascular disease involves the microcirculation and arterioles and comprises swelling of the intima, intimal proliferation in the arterioles and distortion of the capillaries with occasional capillary necrosis. Endothelial apoptosis has been recognized as an important component of the vascular disease [10,11]. The resulting capillary destruction leads to a reduced size of microvascular beds, followed by decreased organ blood flow, eventually resulting in chronic ischemia. In addition, patients with SSc demonstrate a vascular dysfunction that is characterized by vascular permeability, a deregulated control of the vascular tone as well as an activation of the platelets and the coagulation systems [10,12].

Endothelin is a potent vasoconstrictor that is released by fibroblasts [13]. Endothelin overexpression has been associated with various medium- and long-term physiologic processes, such as mitogenesis, fibrosis, vascular hypertrophy, inflammation, and tissue remodeling [14,15]. Additionally, there is now accumulating evidence that endothelin-1 (ET-1) is a key mediator in the regulation of the vascular tone. In SSc, the endothelin production is significantly enhanced, leading to vasoconstriction, vessel remodeling, local ischemia and formation of ulcers of the fingertips [16,17]. So far, bosentan represents the only approved drug for the treatment of SSc-related symptoms, namely digital ulcers.

The treatment of SSc includes the following objectives: reduction of vasospastic phenomena, improvement of vascular permeability, counteracting endothelial dysfunction and antiplatelet action, prevention of visceral involvement, and improvement in quality of life (QOL) [18-21]. New specific therapies have been developed targeting prostacyclin and endothelin, two major mediators governing endothelial function, leading to endothelial dysfunction [1]. In this context, stable analogs of prostacyclin, like iloprost, have shown efficacy and improved life expectancy in patients with SSc [21-23]. The main pharmacological effects of iloprost are inhibition of platelet aggregation and vasodilatation. Both effects are mediated by an activity of adenylate cyclase/cAMP complex, activation of fibrinolysis, and reduced release of free oxygen radicals [24,25].

Bosentan is a dual endothelin receptor antagonist. It competes with ET-1 by binding to the receptors ET-A and ET-B, which are localized in the endothelial and muscle layers of the blood vessel walls. The contribution of ET-1 to the development of digital ulcers and the efficacy of bosentan therapy in patients with SSc was assessed in clinical studies by Korn *et al*. and Matucci-Cerinic *et al*. [26,27]. RAPIDS-2 (RAndomized, Placebo-controlled study on the prevention of Ischemic Digital ulcers secondary to Scleroderma) demonstrated a reduced incidence of new digital ulcers in those patients who already had ulcers, whereas bosentan did not exert any effect on the healing of ulcers [27]. The results of RAPIDS-2 are also included in a recent meta-analysis of the healing and prevention of digital ulcers in patients with SSc by Tingey *et al*. Notably, results from studies on iloprost have been similar, demonstrating no statistically significant effects on the healing or improvement of digital ulcers in patients with SSc, while intravenous iloprost was reported to prevent new ulcers [28]. In spite of the aforementioned positive effects of both bosentan and iloprost in the treatment of SSc, few common, non-serious adverse effects have to be mentioned, including vasodilatation leading to flush, headache, gastrointestinal symptoms, hypotensive reactions, bradycardia or paresthesia. Moreover, bosentan therapy has been associated with an elevation of the liver aminotransferases (ALT and AST) as well as bilirubin [29-31].

Our own clinical observations suggest that the frequency of centrofacial telangiectasia (TAE) may be increased in patients with SSc treated with bosentan. Here, we sought to assess the frequency on TAE in patients with SSc treated with bosentan or iloprost. Results may point toward a hitherto little-known, in some cases stigmatizing adverse effect of bosentan therapy.

## Methods

### Patients

We conducted retrospective analysis in 27 Caucasian patients with SSc (24 female, 3 male; median age 60.9 years, range 27 to 81 years; median disease duration 13.5 years, range 2 to 32 years) treated with either iloprost (n = 16) or bosentan (n = 11). Of these, 17 had lcSSc, 7 had dcSSc and 3 had overlap syndrome, according to the criteria of the American College of Rheumatology (ACR) and LeRoy *et al*. [4]. Patients of the iloprost and bosentan group did not show relevant differences with regard to gender, age, type of diseases, profiles of autoantibodies, or co-medications. The patients’ characteristics are listed in Table 1.

**Table 1:** Characteristics of the subgroups of patients treated with either iloprost (n = 16) or bosentan (n = 11)

**Characteristic**	**Iloprost**	**Bosentan**
lcSSc	12 (75%)	5 (45.5%)
dcSSc	2 (12.5%)	5 (45.5%)
Overlap	2 (12.5%)	1 (9%)
CREST+	8 (50%)	4 (36.4%)
ANA+	14 (87.5%)	11 (100%)
ACA + (overlap excluded)	5 (35.7%)	3 (30%)
Anti-Scl-70 (overlap excluded)	5 (35.7%)	4 (40%)
Anti-CENP-B (overlap excluded)	2 (14.3%)	2 (20%)
Anti-Ro/SS-A (overlap excluded)	4 (28.6%)	1 (10%)
Anri-Ro/SS-B (overlap excluded)	1 (7.1%)	0
Anti-RNP (-Sm, -70) (overlap excluded)	1 (7.1%)	1 (10%)

### Medication

Bosentan (Tracleer®, Actelion Ltd., Allschwil, Switzerland) was administered at a starting dose of 62.5 mg twice daily and increased to 125 mg twice daily after four weeks of treatment according to the manufacturer’s recommendation. Iloprost (Ilomedin®, 20 μg/ml, Schering AG, Berlin, Germany) was given intravenously, body-weight adapted (0.5 ng/kg per minute for eight hours on five successive days), according to the manufacturer’s recommendation. Patients received additional therapy with antimalarial and immunosuppressive agents. Other co-mediations are listed in Table 2.

**Table 2:** Co-medication of the subgroups of patients treated with either iloprost (n = 16) or bosentan (n = 11)

**Co-medication**	**Iloprost**	**Bosentan**
Calcium channel blockers	10 (62.5%)	7 (63.7%)
Acetyl salicylic acid	7 (43.8%)	6 (54.5%)
Nitrates	3 (18.8%)	2 (18.2%)
β-blockers	1 (6.3%)	3 (27.3%)
Diuretics	2 (12.5%)	3 (27.3%)
ACE-inhibitors	2 (12.5%)	2 (18.2%)
Statins	2 (12.5%)	1 (9.1%)
Systemic steroids	5 (31.3%)	4 (36.4%)
Methotrexate	4 (25%)	1 (9.1%)
Azathioprine	0 (0%)	1 (9.1%)
Analgesics	16 (100%)	10 (90.9%)
Antidepressants	4 (25%)	3 (27.3%)
Proton pump inhibitors	15 (93.8%)	10 (90.9%)
Thyroxine	3 (18.8%)	2 (18.2%)
Warfarin	0 (0%)	1 (9.1%)

### Assessment of TAE

Standardized photodocumentation of all patients was performed after ten months of therapy after obtaining informed consent. After we had initially observed a potentially higher incidence of TAE in patients treated with bosentan, new patients referring to our department for bosentan or iloprost therapy were photodocumented also prior to the initiation of therapy. This subgroup (n = 12) did not show any relevant differences (for example. gender, age, disease characteristics, duration of therapy) with regard to the rest of the cohort. For the assessment of TAE, digitized images were edited with monochrome and red-adjustment. All images were obtained and digitized for blinded reading.

### Statistical methods

Data were evaluated using Student’s *t*-tests. For comparing the overall difference for patients treated with bosentan and patients treated with iloprost, a *P*-value from a two-sided Student’s *t*-test was calculated. An addition, multivariate linear regression models with frequency of TAE as response variable and treatment (bosentan versus iloprost) in combination with other clinical factors as explanatory variables were calculated. In order to investigate treatment effects over time within the subgroups of patients for both therapies separately, paired Student’s *t*-tests were conducted. Reported *P*-values for Student’s *t*-tests were corrected for multiple comparisons by considering the conservative Bonferroni correction. *P*-values of less than 0.05 were declared to be significant.

## Results

### Frequency of TAE

**Figure 1:**
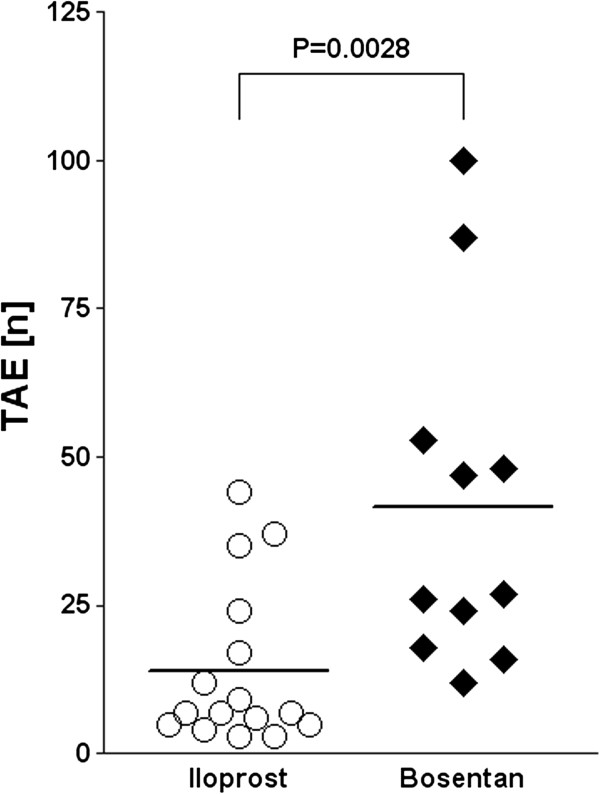
**Frequency of centrofacial telangiectasia (TAE) patients with systemic sclerosis (SSc) treated with bosentan or iloprost over ten months.** Patients receiving bosentan (n = 11) showed a significantly (*P* = 0.0028) higher frequency of centrofacial TAE (n = 41.6 ± 27.8) as compared to TAE in patients receiving iloprost (n = 16; n = 14.8 ± 13.1). Values are plotted as individual ratios, including mean ratio values shown by the horizontal bar. Data were evaluated using a Student’s *t-*test.

**Figure 2:**
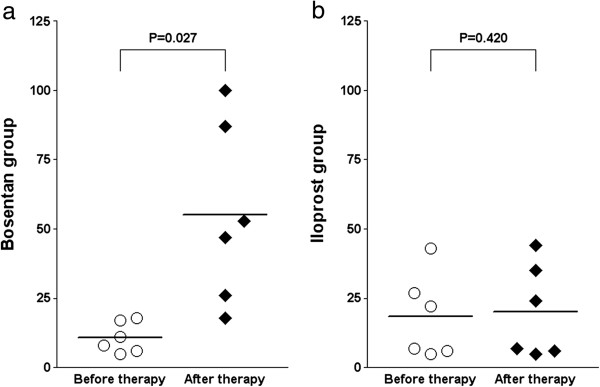
**Development of centrofacial telangiectasia (TAE) in patients with systemic sclerosis (SSc) treated with bosentan or iloprost over ten months.** Patients receiving **(a)** bosentan (n = 6) developed significantly (*P* = 0.027) more TAE (TAE before therapy: n = 10.8 ± 9.5; TAE after ten months of therapy: n = 55.2 ± 29.8) as compared to patients receiving **(b)** iloprost (n = 6; TAE before therapy: n = 18.3 ± 14.5; TAE after ten months of therapy: n = 20.2 ± 15.5; *P* = 0.420). Values are plotted as individual ratios, including mean ratio values shown by the horizontal bar. Data were evaluated using a Student’s *t-*test.

After ten months, patients treated with bosentan (n = 11 patients) showed a significantly (*P* = 0.0028) higher frequency of centrofacial TAE (n = 41.6 ± 27.8) as compared to patients receiving iloprost (n = 16 patients; n = 14.3 ± 13.1) (Figure 1). Multivariate linear regression analyses show that in the presence of other clinically assessed factors, treatment (bosentan versus iloprost) remains the only significant predictor for the frequency of TAE (*P* = 0.099) after adjusting for lcSSc and dcSSc (*P* = 0.017 after adjusting for all clinical factors presented in Table 1). A prospective sub-group analysis of the frequency of TAE prior to and after ten months of therapy demonstrated a significant (*P* = 0.027) increase in the bosentan group (n = 6 patients; TAE before therapy: n = 10.8 ± 5.1; TAE after ten months of therapy: n = 55.2 ± 29.8) (Figure 2a) as compared to the iloprost group (n = 6 patients; TAE before therapy: n = 18.3 ± 14.5; TAE after ten months of therapy: n = 20.2 ± 15.5; *P* = 0.420) (Figure 2b). Whereas the majority of patients in the bosentan group reported a fast or rapid development of TAE after initiation of the bosentan therapy and suspected a likely correlation to the drug, patients of the iloprost group reported a progressive development of TAE over several years and suspected a correlation to the progression of the SSc disease, not the drug.

### Appearance of TAE

**Figure 3:**
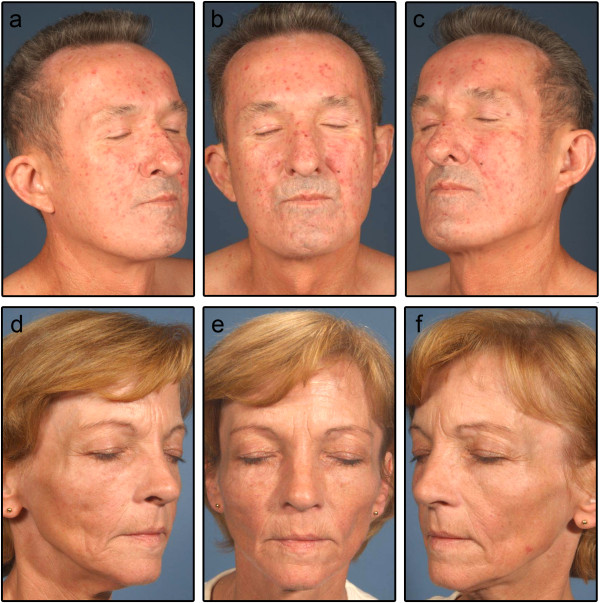
**Clinical appearance centrofacial telangiectasia (TAE) in patients treated with bosentan or iloprost after ten months. (a-c)** 57-year-old man treated with bosentan; **(d-f)** 54-year-old woman treated with iloprost.

**Figure 4:**
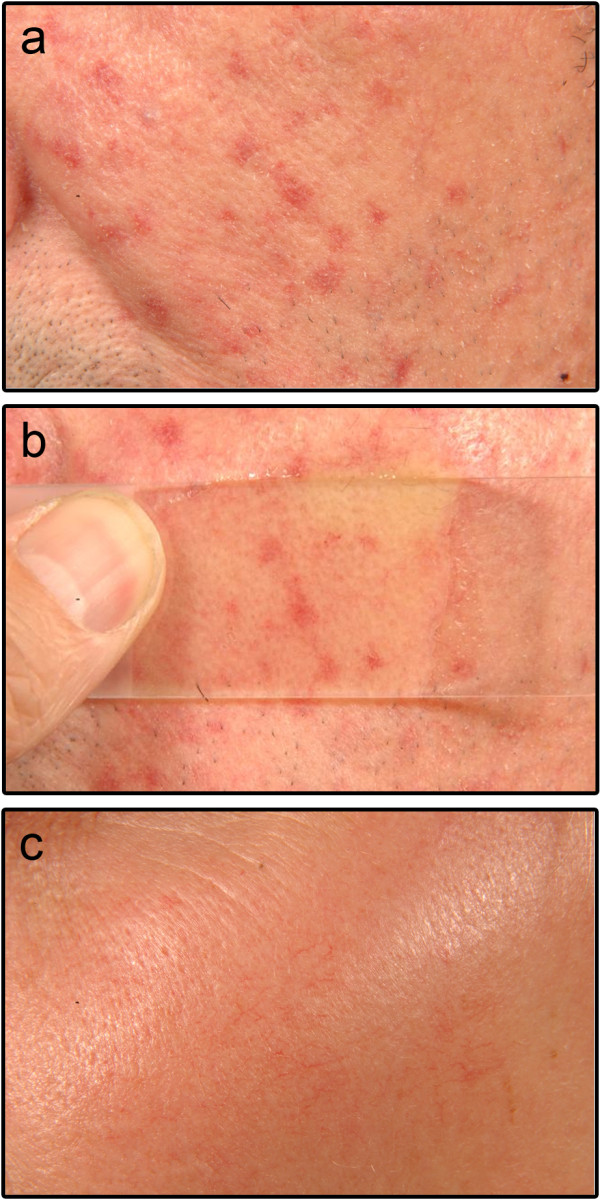
**Clinical appearance centrofacial telangiectasia (TAE) in patients treated with iloprost or bosentan after ten months. (a)** Close-up and **(b)** diascopy of the cheek of a 57-year-old man treated with bosentan; **(c)** close-up of the cheek of 54-year-old woman treated with iloprost.

TAE in bosentan patients had a rather dotted clinical appearance and showed a tendency to persist under diascopic pressure, whereas iloprost patients had rather linear or elongated TAE which tended to fade under diascopic pressure (Figures 3 and 4). The two patients with the most rapid development of TAE discontinued bosentan therapy due to the stigmatizing aspect of the lesions. These two patients did not exhibit any additional special characteristics as compared to the remaining cohort.

## Discussion

The ET-1 receptor antagonist bosentan and the prostacyclin analog iloprost are well established in the management of Raynaud’s phenomenon and ischemic ulcers in patients with SSc [3,25,27,28,32]. The most important documented adverse effects of iloprost include flushing, photosensitivity, jaw pain, headaches, diarrhea, nausea and vomiting [28,33]. Documented adverse effects of bosentan comprise pruritus, urticaria, leukocytoclastic vasculitis, indurated erythema, flushing, peripheral edema, elevated aminotransferases, headache, dizziness, cough, nasal congestion and a potential worsening of symptoms in heart failure patients [27,28,34-37]. Interestingly, even though flushing is a known adverse effect of bosentan, persistent alterations of the facial vasculature such as TAE, have remained largely unnoticed.

TAE are a characteristic feature of connective tissue diseases such as SSc, dermatomyositis and overlap syndromes [4,38]. Indeed, they reflect one of the cardinal symptoms of CREST syndrome (Calcinosis, Raynaud’s phenomenon, Esophageal dysmotility, Sclerodactyly, TAE). Whereas some authors state that the acronym CREST is obsolete, others still considered it to be a form of a limited cutaneous SSc (lcSSc) [2,39]. Hence, the observation of TAE in our cohort of patients with SSc is not surprising. Yet, while it cannot be ruled out that the increase in the number of TAE is a consequence of a worsening of the disease over the course of the therapy, the frequency and rapid progression of TAE in patients with SSc treated with bosentan is remarkable. This hypothesis is supported by the fact that patients treated with bosentan suspected that the onset of TAE correlated to the administration of the drug, whereas no such correlations were suspected by patients treated with iloprost. A further limitation of our study is the small number of patients included. Yet, our results are in line with a recent case report by Tong and Kumarasinghe in a 76-year-old woman treated with bosentan for four years. In this patient, a prominent flushing gradually progressed to persistent redness and TAE [37].

The molecular and cellular mechanisms governing the development of TAE in SSc, as well as the mechanisms by which bosentan may induce persistent vascular alterations, have remained largely elusive. It has been proposed that TAE in SSc develop as a response to endothelial injury. This concept is supported by micro-capillaroscopic analyses that reveal an extensive derangement and destruction of the microvasculature in a variety of organ systems [38]. Interestingly, the distribution and appearance of TAE in SSc correspond to TAE in patients with hereditary hemorrhagic telangiectasia (HHT; Osler-Weber-Rendu syndrome), pointing toward similar pathogenetic mechanisms. HHT is an autosomal dominant disorder of the vasculature development characterized by TAE and arteriovenous malformations [40]. Abnormal TGF-β signaling has been shown to play a crucial role in the pathogenesis of HHT [41]. Moreover, TGF-β signaling has been recognized as a key regulator of wound healing and fibrosis and exerts a variety of effects on the biology of endothelial cells and vascular tissue [1]. Likewise van Royen *et al*. could show that exogenous TGF-β stimulated the angiogenesis in the peripheral circulation in an *in vivo* rabbit model [42]. Interestingly, patients with SSc show elevated serum levels of connective tissue growth factor (CTGF), a downstream target of TGF-β, and scleroderma fibroblasts show an increased expression of the TGF-β receptor [43,44]. Therefore, it is tempting to speculate that TGF-β signaling may also play a role in the pathogenesis of TAE in patients with SSc [38].

With regard to ET-1 antagonists, a recent case report demonstrated a significant alteration of the macrovascular involvement by bosentan in a 50-year-old Japanese patient with SSc. Magnetic resonance angiography showed an attenuation of a stenosis of the ulnar artery. The authors concluded that bosentan, besides reversing the vasoconstrictive effects of ET-1, also exerts remodeling effects on the vasculature [45]. Accordingly, ET-1 has been shown to contribute to the mitogenic activity of fibroblasts and smooth muscle cells *in vitro*[46,47]. Hence, the promotion of TAE development by bosentan in patients with SSc may be the result of vasodilatatory and/or direct vascular remodeling effects.

## Conclusions

In summary, we here show that bosentan therapy may be associated with a significant increase in the number of facial TAE. Because these stigmatizing lesions are a potential obstacle to patients’ adherence to therapy, they should be informed about this adverse effect that has remained largely unrecognized until recently. Management options may include camouflage ointment or laser therapy.

## Abbreviations

ANA: Antinuclear antibodies; ACA: Anti-centromere antibodies; ACR: American College of Rheumatology; CREST: Calcinosis Raynaud’s Esophageal dysmotility Sclerodactyly Telangiectasia; CTGF: connective tissue growth factor; ET-1: endothelin-1; HHT: hereditary hemorrhagic telangiectasia; QOL: quality of life; SSc: systemic sclerosis, scleroderma; lcSSc: limited cutaneous scleroderma; dcSSc: diffuse cutaneous scleroderma; RAPIDS: Randomized, Placebo-controlled study on the Prevention of Ischemic Digital Ulcers secondary to Scleroderma; TAE: telangiectasia.

## Competing interest

PAG and SM have received travel/meeting support by Actelion Ltd., Allschwil, Switzerland.

SM has received research funding by Actelion Ltd., Allschwil, Switzerland.

## Authors’ contributions

SH collected the data. BAB, HS, EB, SM, PAG and BH performed data analysis and interpretation. KK performed statistical analyses. BAB, PAG and BH wrote the manuscript. All authors read and approved the final manuscript.
